# Ambient Intelligence Environment for Home Cognitive Telerehabilitation

**DOI:** 10.3390/s18113671

**Published:** 2018-10-29

**Authors:** Miguel Oliver, Miguel A. Teruel, José Pascual Molina, Dulce Romero-Ayuso, Pascual González

**Affiliations:** 1 LoUISE Research Group, Research Institute of Informatics, University of Castilla-La Mancha, 02071 Albacete, Spain; oliver@dsi.uclm.es (M.O.); miguel@dsi.uclm.es (M.A.T.); 2 LoUISE Research Group, Computing Systems Department, University of Castilla-La Mancha, 02071 Albacete, Spain; JosePascual.Molina@uclm.es; 3 Department of Physical Therapy, Occupational Therapy Division, Faculty of Health Sciences, University of Granada, 18016 Granada, Spain; dulceromero@ugr.es; 4 CIBERSAM, Biomedical Research Networking Centre in Mental Health, 28029 Madrid, Spain

**Keywords:** computer-assisted telerehabilitation, pervasive computing, ambient assisted living, wearable sensor, electroencephalogram (EEG) headset, Kinect, haptic stimulus, fuzzy inference system, distributed system, serious game

## Abstract

Higher life expectancy is increasing the number of age-related cognitive impairment cases. It is also relevant, as some authors claim, that physical exercise may be considered as an adjunctive therapy to improve cognition and memory after strokes. Thus, the integration of physical and cognitive therapies could offer potential benefits. In addition, in general these therapies are usually considered boring, so it is important to include some features that improve the motivation of patients. As a result, computer-assisted cognitive rehabilitation systems and serious games for health are more and more present. In order to achieve a continuous, efficient and sustainable rehabilitation of patients, they will have to be carried out as part of the rehabilitation in their own home. However, current home systems lack the therapist’s presence, and this leads to two major challenges for such systems. First, they need sensors and actuators that compensate for the absence of the therapist’s eyes and hands. Second, the system needs to capture and apply the therapist’s expertise. With this aim, and based on our previous proposals, we propose an ambient intelligence environment for cognitive rehabilitation at home, combining physical and cognitive activities, by implementing a Fuzzy Inference System (FIS) that gathers, as far as possible, the knowledge of a rehabilitation expert. Moreover, smart sensors and actuators will attempt to make up for the absence of the therapist. Furthermore, the proposed system will feature a remote monitoring tool, so that the therapist can supervise the patients’ exercises. Finally, an evaluation will be presented where experts in the rehabilitation field showed their satisfaction with the proposed system.

## 1. Introduction

One of humankind’s major aims is to extend life expectancy. Thanks to studies and efforts by the research community, human beings are living a little longer every day [1]. However, this has brought a consequence that affects people’s quality of life, namely increasingly-common cognitive problems due to aging. Because of this, our goal has changed from living longer to living longer and better. In this context, rehabilitation is one method for achieving this goal. It is defined by the World Health Organization [2] as an ”active process by which those affected by injury or disease achieve a full recovery or, if a full recovery is not possible, realize their optimal physical, mental and social potential and are integrated into their most appropriate environment”. Rehabilitation comprises an extensive variety of activities and services intended at reducing the impact of injuries and disabilities by applying different processes and strategies.

There are some computer-assisted cognitive rehabilitation systems that through the use of serious games (“games in which education in its various forms is the primary goal rather than entertainment” [3]) try, to some extent, to correct one or more of patients’ cognitive impairments. The serious gaming approach in system development allows users of such systems to feel more motivated, and try to get better results in the best possible way [4]. However, traditional cognitive rehabilitation systems are sometimes not effective enough. As Hotting and Roder have pointed out [5] “physical exercise may trigger processes facilitating neuroplasticity and, thereby, enhances an individual’s capacity to respond to new demands with behavioral adaptations”. In the same direction, Wimstein et al. [6] noted that exercise may be considered as adjunctive therapy to improve cognition and memory after stroke. Thus, the combination of physical and cognitive training might result in a mutual enhancement of both interventions. In fact, the performance of activities of daily living requires the patient to integrate physical and cognitive abilities and skills simultaneously [7]. 

Furthermore, due to the treatments’ length, it is frequently necessary that patients carry out some rehabilitation exercises at home for improving their lost capabilities. This is the objective of telerehabilitation systems [8]. As Anton et al. point out [9], these systems must provide therapists with the possibility of selecting the most appropriated therapies for the patient, evaluating their execution and managing them in a remote manner. In this case, the patients’ monitoring is a very relevant and complex task, since, in general, the therapist is not present. Thus, the use of any physiological sensor for monitoring the physical and psychological conditions of the patients is paramount in our target systems. Moreover, together with patients’ remote monitoring, the inclusion of multi-sensory and adaptation features are also a cornerstone in the next generation of telerehabilitation systems [10]. 

We propose an ambient intelligence environment for cognitive telerehabilitation system that enables the therapist to design exercises combining physical and cognitive activities, as in the real world, and facilitates the achievement of greater personal independence [11]. Our solution also monitors the patient’s activity and provides help when needed. Moreover, it also guides the execution of the next steps within the rehabilitation session. This last feature is achieved by implementing a Mamdani Fuzzy Inference System (FIS) [12] that simulates the knowledge of the rehabilitation specialist. Thus, it takes input not only from the patient’s performance on the exercise, but also from his/her stress level, thanks to sensors such as EEG headsets. Hence, the patient will be able to carry out the exercises without the need for constant supervision of the therapist. The choose of stress as the physiological signal to be monitored is because this is a normal response when someone undertakes activities that are beyond the person’s level of competence [13]. In addition, as Dinse et al. point out [14], stress-related mechanisms inhibit perceptual learning in a similar manner to episodic memory retrieval [15]. Thus, we have considered that the presence of high stress levels in the users when they are executing a cognitive rehabilitation exercise could affect task performance negatively, and, therefore, it must be considered as an input variable that should be included in our FIS.

Besides the sensors included in our proposal, the use of haptic (vibrotactile) actuators [16] provides support to the patient when carrying out some exercise, offering a multi-sensory experience, since it complements the visual and aural channels with other stimuli. Our proposal, which is based on our previous works [17,18], introduces as a new feature the use of stress sensors, haptic actuators and the inclusion of a FIS for controlling the exercise execution, which are used to replace, somehow, the therapist supervision and support when the patient carries out the exercises. This gives more autonomy to the therapist who can design exercises, monitor patients, and diagnose errors in the execution of exercises, from anywhere at any time. It will also increase the independence of patients, who will be able to carry out rehabilitation exercises from their own homes, together with their relatives or caregivers, as well as reducing the cost and improving their rehabilitation process.

The content of this paper is structured as follows: next, in Section 2, an introduction on assistive technologies and their use in cognitive rehabilitation therapies will be presented. After that, in Section 3, we will show our proposal of an ambient intelligence system for cognitive telerehabilitation, combining physical and cognitive activities, at home. For this purpose, in Section 4, Section 5 and Section 6, we will begin by presenting the sensors used, the remote exercise viewer, and the diffuse inference engine that will give the system its autonomy. In addition, Section 7 will show the evaluation of the system by rehabilitation experts. Finally, in Section 8, the conclusions reached during the development of this system will be discussed.

## 2. Cognitive Rehabilitation Technologies

There are some computer-assisted cognitive rehabilitation systems that aim to help different groups of people to overcome cognitive impairment. For example, Franco-Martin et al. [19] attempted to improve patients suffering from traumatic brain injury, schizophrenia or dementia. For this purpose, they made use of preset exercises that are divided into attention, perception, memory, calculation, orientation, language and reasoning modalities. JClic [20] is another example of software for the development of multimedia activities to assist in the education of primary school children, but that is also used for the rehabilitation of cognitive impairment.

One of the commonest sensors in physical rehabilitation systems is a depth camera such as the Microsoft Kinect [21]. However, in cognitive rehabilitation many different sensors are used, although there are some proposals that integrate the gesture recognition capability of the Kinect to control some cognitive rehabilitation tasks. One of these systems is the Tangible Goals:Health (Tango:H) system [22]. It focuses on the rehabilitation of hospitalized children with cognitive disabilities, with the creation of social active play with gestural interaction. This system uses a Kinect depth camera that captures the body shape of the patients and it is based on interaction with the patient’s own-body gestures. The use of Kinect depth cameras as a device to capture patient movements in cognitive rehabilitation tasks was assessed by Cogollor et al. [23] too. These authors concluded that the Kinect sensor offers results that are acceptable enough for use in this type of task.

In addition, there are other rehabilitation systems that use different sensors and/or actuators. For example, the system proposed by Teruel et. al [24] eases the rehabilitation of people with hemispatial neglect. This proposal uses multisensory stimuli (vision, hearing and touch) to assist patients in their rehabilitation process. With this aim, it makes use of a virtual reality headset (3D vision + 3D sound) and a haptic (touch) stimulation device. Other proposals, such as the one presented by Yu et al. [25], also use systems featuring different stimuli to help patients in their rehabilitation process. They present the fusion of data from the Leap Motion camera and data from an omega.7 device for the rehabilitation of bilateral complementary coordination.

The main problem with this kind of systems is the need for constant supervision by a therapist. The rehabilitation specialist must monitor in real-time the progress of the exercise as well as the patient’s current condition. Hence, the therapist will be able to adapt the patients’ cognitive load to their current state, decreasing the difficulty if the patient is having problem while performing the exercise, or even canceling the exercise. One way to avoid the need for constant supervision by a therapist is to use a Fuzzy Inference System (FIS), as we propose in this paper. A FIS is a system that takes input variables and maps them into an output variable using fuzzy logic techniques. This enables the creation of models based on fuzzy rules that control the execution of a program. The main advantage of this type of system is the simplicity it offers when creating the knowledge base, which enables people without prior knowledge to design and create rules that model specific behavior bases on their own knowledge. This simplicity is achieved because the rules are built in an If-Then format based on natural language.

The use of Fuzzy Inference Systems in the rehabilitation domain is not a novelty, especially in physical rehabilitation systems. An example of rehabilitation solution proposed in the literature is the one presented by Zhang et al. [26]. This physical rehabilitation system was designed to deal with the upper limbs of people after a stroke. The objective of the FIS presented in this work is to evaluate the movement of the upper extremities. To measure these movements, they use a device attached to the patient’s body that gathers acceleration data from such limbs. Besides, the system presented by Gal et al. [27] uses a Kinect and a FIS for physical rehabilitation of patients. In this case, the FIS is used to detect the patient’s posture, and to evaluate it depending on the diffuse rules implemented. If the position adopted by the patient entailed some risk, it will be notified visually. Another system that makes use of a FIS is the one presented by Su et al. [28]. This system enables, using Kinect sensors, the physical rehabilitation of patients in their own home. In this case, the FIS is used to evaluate the rehabilitation results by comparing data about the speed, trajectory and performance that the user obtains at home, with data obtained in a rehabilitation center under the supervision of a specialist. These examples use a FIS in the physical rehabilitation domain, but they do not consider other problems related to cognitive rehabilitation that a user faces when performing an exercise at home. Thus, for improving the support of the patients when carrying out a therapy at home, we have included some sensors and actuators that could be considered as the therapist senses for monitoring the exercise and they’ll try to act like the therapist would. For that propose, the information provided by the sensors are processed by our intelligence system (FIS) and it provide the most adequate feedback, by using the visual channel or including other senses by means of the haptic actuators. 

Thus, for developing an intelligent system (FIS) that helps the telerehabilitation system to make decisions, it is necessary that the system controls the patient’s physical and mental state. As Panja et al. [29] stated: “Biomedical signal processing plays a major role in developing a home rehabilitation system”. Because of this, in their work they propose the use of different sources to gather the patient’s physiological state (electrocardiogram (ECG), electromyogram (EMG) and electroencephalogram (EEG)) for possible use in telerehabilitation systems. Rodriguez et al. [30] presented a FIS that enables using some physiological variables, such as fatigue and performance, for controlling the evolution of the exercises of physical rehabilitation proposed by the therapist. This proposal defines some conceptual models to describe the use of FIS in rehabilitation process in the adaptation of the exercise difficulty.

## 3. Ambient Intelligence Environment for Cognitive Telerehabilitation

This system is an extension of the one presented in previous works such as [17,18]. In the first one [17], we included some relevant social features to improve motivation and reduce the feeling of social isolation. This proposal provides the opportunity to design multiuser therapies offering a more entertaining environment. The second one [18], presented a first prototype of the physical-cognitive rehabilitation system, where both therapist and patient are in the same physical location. As it can be seen in the previous works, our system combines cognitive and physical rehabilitation by associating the cognitive activity with a physical exercise. This activity is monitored by a Kinect that tracks the physical movements of the patient. This combination of physical and cognitive activities enables motivating the patients in their rehabilitation making these activities more engaging. Moreover, it tries to achieve the advantages of combining these activities, as pointed out by Hotting and Roder [5]. Now, in this paper we present a new version that enables carrying out the cognitive telerehabilitation at home including a deep description of the FIS, sensors and actuators used in the system.

**Figure 1:**
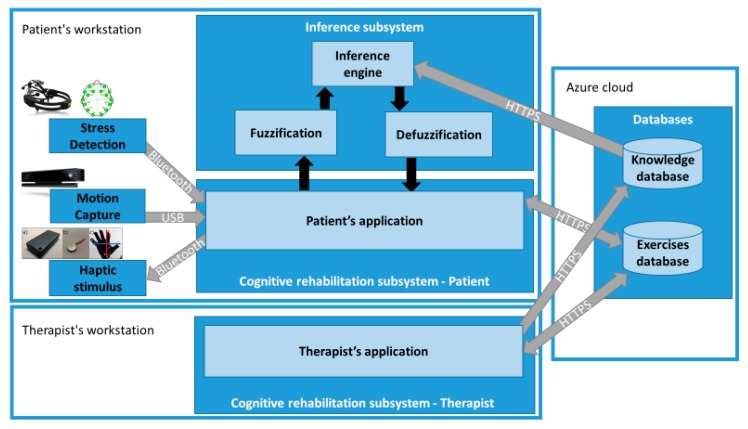
Architecture of the ambient intelligence environment for cognitive telerehabilitation.

Regarding the architecture of the proposed system, it consists of two subsystems: the “Cognitive rehabilitation subsystem” and the “Inference subsystem” (Figure 1). The cognitive rehabilitation subsystem is located on the patient’s workstation, and on the therapist’s workstation. On the one hand, on the therapist’s side, he/she can manage the design of the rehabilitation exercises, manage patients, manage rules of the inference engine, as well as check the progress of rehabilitation exercises performed by patients. On the other hand, the patient’s side is responsible for enabling the patients to perform the rehabilitation exercises. For this purpose, the cognitive rehabilitation subsystem retrieves the rehabilitation exercises that have been assigned to the patients by the therapist, establishes the communication with the sensors and actuators, and interacts with the inference system to adapt the rehabilitation exercises to fit the patient’s needs at the time of execution. 

To alleviate the problem of constant supervision that we have pointed out in other systems, our proposal implements an “Inference subsystem” to determine whether the patients are having problems in the exercise or they could suffer from a high stress level. This new component would determine when it is necessary to decrease the difficulty of the exercise, offering help to the patient, or directly cancelling the execution of the exercise. This decision system facilitates the cognitive rehabilitation system to run in a more independent manner, without the direct supervision of the therapist. Hence, the patient can perform rehabilitation exercises by using the “Patient’s application” from home. It can be achieved with some supervision of people without technical knowledge in rehabilitation, such as the patient’s relatives or caregivers.

### 3.1. Therapist’s Application

The therapist’s application enables the therapist to create exercises, register patients, assign exercises to patients and verify the execution of the exercises completed by them. The exercises created can be based on well-known rehabilitation therapies and be adapted to use them with our depth-camera-based rehabilitation system.

**Figure 2:**
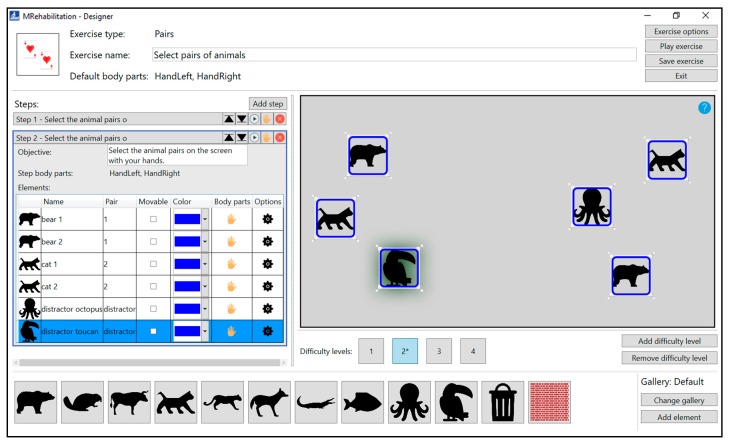
Exercise creation interface.

Figure 2 illustrates a case where the therapist is designing a rehabilitation exercise in which the patient must select the pairs of elements with his/her hands. The exercise is divided in several steps, with the interface showing the second one. This interface, as well as the method for creating rehabilitation exercises, has been explained in our previous works [17,18]. As a new part of the interface, the section to manage the difficulty levels is shown. This section will be used by the FIS to increase or decrease the difficulty of each of the steps when performed by a patient. 

For this step, the therapist has created four difficulty levels. The difficulty of each level will be determined by the number of elements that the patient must select, as well as the number of distracting elements (distractors) that each level contains. The higher the number of elements, the higher the cognitive load that the patient must face when performing the exercise. The difficulty levels created in our rehabilitation proposal will be built upon each other. That is, each difficulty level will have the same elements as its lower difficulty level, as well as one or more new elements that add complexity to it.

### 3.2. Help for the Patient

There are two types of support in our system that can help patients when they have a problem performing an exercise, and therefore they can help the patients to perform rehabilitation sessions from their own home. These aids are visual and vibrotactile.

- Visual help makes it possible for the patient to see which items must be selected. In this case, the elements will increase and decrease their size noticeably, which enables the patient to distinguish them clearly from the rest of the elements.
- Vibrotactile help makes it possible for the patient to feel which way the hand should move to find the next item to activate. This is accomplished by the VITAKI (VIbroTActile toolKIt) device (LoUISE Research Group, Albacete, Spain) [16], which will be explained in more detail in Section 4.3.

### 3.3. Difficulty Levels

**Figure 3:**
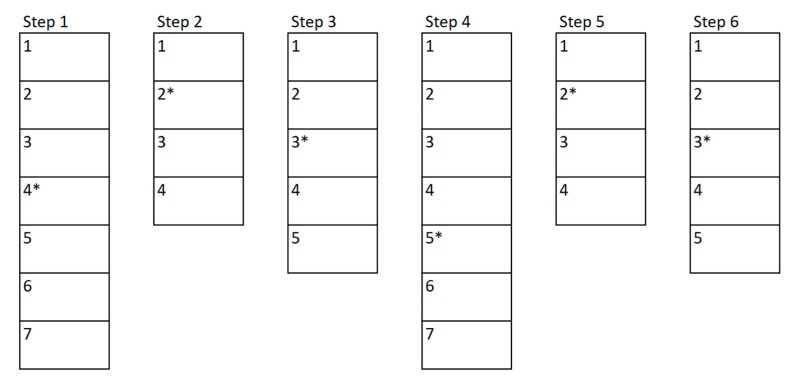
An exercise consists of several steps, and each step, in turn, consist of difficulty levels.

**Figure 4:**
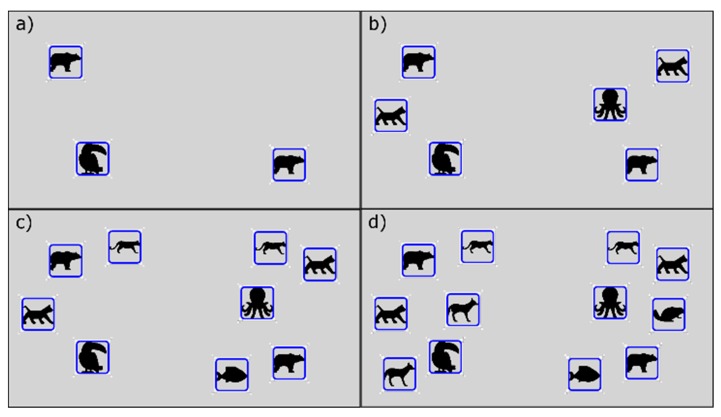
Levels of difficulty in step 2 of the example exercise.

To create an exercise, a therapist must define different steps for the patient to go through them to complete such exercise, as shown in Figure 3. Within each of these steps, the therapist will add levels of difficulty that will make the step increasingly difficult, as illustrated in Figure 4. The therapist will begin to create the first level of difficulty with a blank screen on which to add different elements to be selected or elements that act as distractors. Once he/she thinks the difficulty level is complete, he/she can add a new level. This will create a new difficulty level in which all the elements that were in the previous difficulty level will appear. Previously positioned elements may not be modified, removed, or changed. 

This method enables the therapist to create the difficulty levels incrementally. The higher number of elements, both valid and distractors, the more cognitive complexity that the patient will have to face when performing the exercise. In addition, the therapist should select a level of difficulty that the patient should begin with. This level will be shown to the patient the first time, and according to the performance of the previous step, the FIS system will select which level he should go on to the next step. This will be shown in more detail in the section dedicated to the inference system (Section 6).

For example, Figure 3 shows a possible exercise created by a therapist. The therapist has created a total of six steps to be performed by the patient, these steps are shown in the example as the vertical rectangles. Each of these rectangles is divided into different sub-rectangles indicating the difficulty levels of each step. In the first step there are a total of seven difficulty levels, whilst in the second one there are only four levels. The asterisk shown next to the level number represents the “initial level” that the therapist has considered suitable to be the base level to begin with. In the example, the therapist has set level four for the first step and level two for the second step. Figure 4 shows the example of the different levels related to the second step created by a therapist. This step has a total of 4 incremental difficulty levels. Figure 4a–d shows the difficulty levels increasingly. In this example, it can be seen how the therapist can easily create the levels of difficulty that make up each of the steps of the exercise. 

### 3.4. Patient’s Application

The development of the patient interface has been based on the work proposed by Jaquero et al. [31], more specifically in the guideline “Avoid awareness overloading”. This recommendation refers to the fact that it is not good for the effective understanding of the interface to add all the possible elements of awareness, being these elements the ones that provide information about the system itself, or its users. Although this provides an interface with more information, it is often ignored by the user due to the high cognitive burden of processing it. In this sense, the elements shown to the user have been reduced as much as possible, to reduce the cognitive load that the processing of the interface elements implies for the users. This is of relevance, since in this specific case, the users of the system are patients with cognitive deficits.

**Figure 5:**
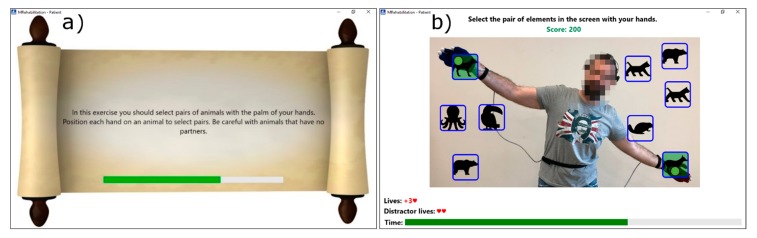
(**a**) Explanation of the exercise shown to the user at the beginning; (**b**) Patient performing a rehabilitation exercise.

At the beginning of the rehabilitation exercise, an explanatory text is displayed with the aim of informing the patient about how to perform the exercise. A screenshot of this interface is shown in Figure 5a. This text should contain enough information for the user to know what to do to complete the exercise successfully. This text is written by the therapist when he/she creates the exercise and also, he/she sets how long this text will be displayed.

Once the intro screen is displayed, an interface like the image of Figure 5b will be shown. A brief explanatory text appears at the top of the interface. This text serves as a guide for the user during the rehabilitation exercise execution. At the bottom, the patient’s current “Score” is displayed. Such score is increased when valid objects are selected and decreased when errors are made. As mentioned above and as Werbach and Hunter point out [4], this kind of gamification motivates the user to obtain a higher score and to perform the exercise in the best possible manner. The exercise itself appears in the central part of the interface. This interface shows the objects placed by the therapist, the patient’s interaction points (by default these points are his/her right and left hand), and the image captured by the camera. In addition to these user-interface objects, the therapist can display more elements through exercise options. With this configuration, it is possible to display exactly the information currently required by the patient to carry out the exercise.

## 4. Sensors and Actuators

Together with a Kinect sensor, used to track the patient’s movements, our system makes use of other sensors to capture some physiological signals that enable the system to estimate when the patient is under a stressful situation. A person’s stress can be measured by electroencephalography, electrocardiography, galvanic skin response, electrodermal activity (EDA), photoplethysmography, electromyography or piezoelectricity/electromagnetic generation [32]. From these, we first consider using electrodermal activity. An example of a sensor that makes use of the EDA to obtain this physiological measure is the E4 wristband [33]. One of the advantages of this sensor is ease of use, just placing it on the wrist of the user and the sensor is able to pick up the level of stress. Also, its comfort is greater than the vast majority of sensors for this task. However, as Gjoreski et al. pointed out [34], physical activity, such as a rehabilitation exercise, can produce an EDA signal that is similar to stress, and so the physiological response could be mistaken. For this reason, the electroencephalography method, which is not affected by the physical activity of the users, was used in the final proposal. The sensor we then selected was the Emotiv headset [35], which will be presented in Section 4.2. In addition to the use of this sensors, our proposal includes a haptic device (VITAKI platform) [16] which provides the patient with haptic sensations through several haptic actuators (vibrotactile actuators). In this case, the patient feels in which the direction the hand should be moved to find the next item. The limitation of the sensors is the cost that these present. Although the cost of the sensors can be around 1000$, this is not very large compared to other devices and proposals in the field of rehabilitation of patients. But this limitation must be taken into account if the system will be deployed. In the following, the sensors and actuators used in our home rehabilitation proposal are presented. Moreover, their characteristics, how they work within the system, and their limitations are presented.

### 4.1. Microsoft Kinect v2

**Figure 6:**
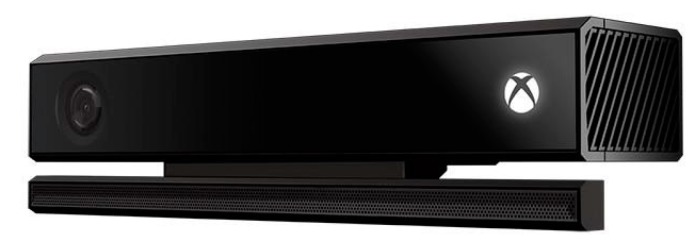
Microsoft Kinect v2 sensor (Microsoft Corp., Redmond, WA, USA).

This low-cost device, shown in Figure 6, enables the recognition of people and objects in three dimensions by capturing three-dimensional images. The sensors available are (i) a color camera that captures the image of the environment in high resolution at 1920 × 1080 @ 30 fps, and (ii) a Time-of-Flight depth camera that enables to obtain the distance from the sensor to the objects and captures the image of the environment at 640 × 480 @ 30 fps. People recognition with this device works between 0.8 to 4 m away from the sensor. This sensor is connected to the computer via a USB 3.0 port and enables people to interact with the computer without having to make physical contact with it or with another device [21].

The only limitation of this sensor is the need to capture the patient’s image, so the patient must be informed and agree to the use of cameras that record their rehabilitation therapy.

### 4.2. Emotiv Epoc+

The placement of this headset is fundamental, and the measures taken by the sensor depends on whether the placement are correct or not. Because of this, before starting the exercise, and during the whole course of it, the system will check the correct placement of the headset, and in the event that the electrodes move, and do not allow a proper reading, will warn the user, family or caregivers. The warning will be visual and will indicate that the electrode is not correctly positioned, allowing an easy and fast way to adjust the electrode.

**Figure 7:**
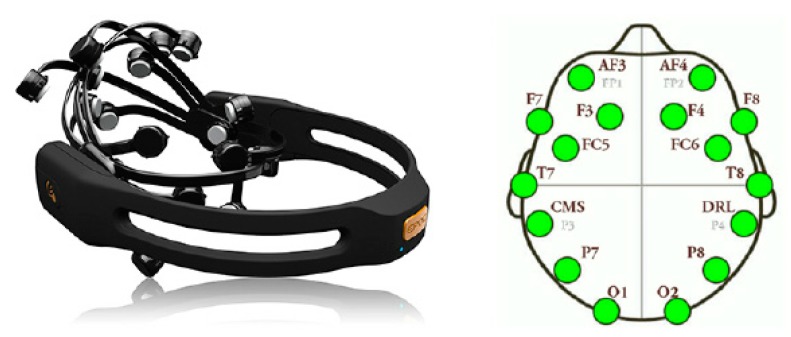
Emotiv Epoc+ headset and electrodes location (Emotiv, San Francisco, CA, USA).

This device is a 14-channel electroencephalogram (EEG) headset, with 14 or 16 bits of resolution per channel, widely used for research purposes due to its price range (~800 $), this headset is shown in Figure 7 together with the placement of the electrodes on the user’s head. Thanks to this peripheral, it is possible to get (i) performance metrics, i.e., six different subjective emotions experienced by the user (engagement, interest, focus, excitement, stress and enjoyment) (ii) mental commands, i.e., real-time brainwave of the user for discerning his/her attempt to perform physical actions over objects (either physical or virtual ones), (iii) facial expressions (blink, wink, smile, clench, smirk, laugh, and so on) and (iv) head position thanks to a 2-axis gyroscope. Through a wireless connection it enables the system to obtain the metrics of the user’s brain activity, avoiding the risk of entanglement with wires during the rehabilitation exercises. This gives great autonomy of movement to the patients. The design of this device enables its placement and use by non-expert users, which facilitates its use by patients of the rehabilitation system [36].

There are two limitations on the use of this headset. The first limitation is the need to place it on the user’s head, and the second limitation is the accuracy and errors in the measurements taken. The first limitation can make the system unusable by patients in their own home if they are unable to put on the headset properly. For this reason, it is necessary that in cases where the patient is unable to perform this task, he/she must be accompanied by relatives or caregivers. After being instructed in the use of the headset, they will assist the patient to place the headset in order to carry on the rehabilitation process. The second limitation relates to the precision of the headset to measure the patient’s stress. While this may seem like a major limitation, there are studies that have determined the accuracy of this sensor can be up to 88–96% when using a two-level classification [37]. In addition, to reduce any possible erroneous readings provided by the headset, an algorithm is used to smooth any abrupt changes in the readings. This algorithm will be presented in Section 6.1.1.

### 4.3. VITAKI

This device has been developed in our laboratory for haptic stimulation. VITAKI has eight outputs to which eight vibrotactile actuators can be connected, enabling us to activate/deactivate them independently. Hence, the computer will send the haptic stimuli via Bluetooth to VITAKI, which will receive the packets with the information and process them to send the appropriate voltage to the vibrotactile actuators. These vibrators will produce the vibrotactile stimulus that the user will receive on his/her skin. This device has been used, among other things, to identify 3D virtual objects [38] or weight and size discrimination [39] by using gloves with vibrotactile actuators. These actuators can be placed over the patient’s hand to make them feel the objects in the rehabilitation environment.

**Figure 8:**
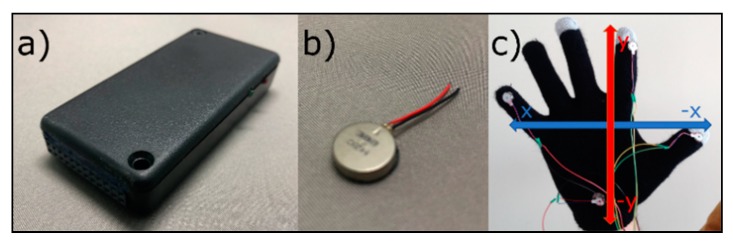
(**a**) Vibrotactile device VITAKI; (**b**) Vibrotactile actuator used by VITAKI; (**c**) Coordinate axis in the user’s palm formed by four vibrotactile actuators.

In our rehabilitation system, this device is used to help patients find the item(s). This is achieved through the Vitaki platform [16] (shown in the Figure 8a), which produces a haptic stimulus in the user’s skin, using vibrotactile actuators (shown in the Figure 8b). This stimulation is placed on the thumb, index and pinky fingers, as well as on the palm of the hand, which provides a virtual coordinate-axis-like guidance in the patient’s hand. These coordinate axes, illustrated in Figure 8c, indicate whether the patient should move the hand to the right, left, up or down depending the actuator that is turned on. 

The limitation of this actuator, as in the previous case, is the possible difficulty to be placed by the patients on his/her body. This limitation has been addressed from two different ways. On the one hand, it is proposed to be placed on the patient’s body by relatives or caregivers, as was the case with the Emotiv headset. On the other hand, we are investigating the improvement of the device to make it easier to wear, making the whole system integrated in a glove.

## 5. Remote Exercise Viewer

Once a patient has performed a repetition of an exercise, it will be sent and stored on the Azure cloud server [40] with the purpose of being monitored by the therapist. In this sense, both the therapist and the patient can perform their functions without the need of being physically together.

The patient’s cognitive rehabilitation subsystem will capture the exercise data as well as the patient’s data at one-second intervals from the moment the patient begins to perform the exercise. According to rehabilitation specialists, this one-second interval is small enough not to lose information among samples on cognitive rehabilitation. Moreover, such interval is big enough to avoid unnecessary cost overruns with possibly irrelevant information. It is worth noting that the patient’s rehabilitation environment may be his/her own home, where a low-speed internet connection or even a limited-data connection may have to be used for cloud database communication purposes. The data gathered in each sample is the relevant information related to the design of the exercise, the execution and the parameters sent to the inference engine. This information is presented in the following:

- Exercise options: Repetitions; Type of help; Maximum exercise time; Cognitive lives; Distractor (elements added to increase cognitive load) lives; Initial explanation; and Initial explanation time.
- Step data: Percent completed; Activated elements; Cognitive errors; Distractor errors; and Elapsed time.
- FIS Input data: Current stress; Time without interaction; Consecutive errors; Step average stress; Step errors ratio; Step canceled.

This data accurately reflects what is happening during the exercise and is provided to the therapist through the remote interface of the cognitive rehabilitation system, so that he/she can monitor it wherever and whenever he/she wants. In addition to the numerical data, a screenshot of the patient performing the exercise will also be saved, so that the therapist has all the information when supervising it.

**Figure 9:**
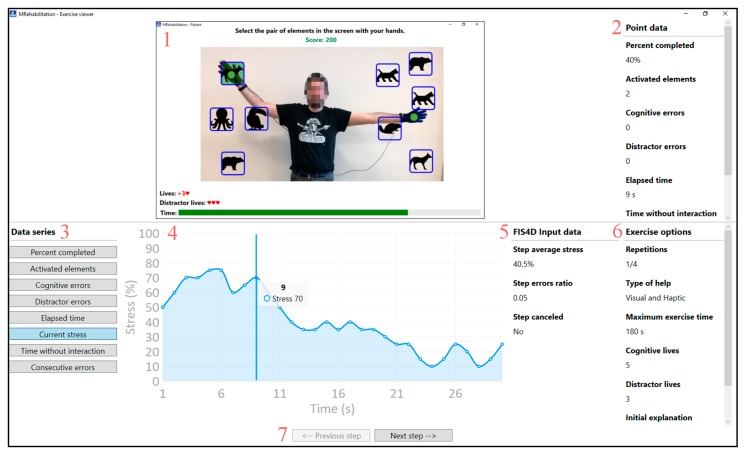
Remote exercise viewer.

For this purpose, the therapist must select the patient, the exercise, as well as the repetition that he/she wants to supervise. This will be display in the monitoring interface as shown in Figure 9. This figure is divided into two different areas. The upper part displays the information about a specific moment in the exercise, and the lower part displays the overall information about the exercise and the current step. The top-central part (1) shows the interface that the patient sees when performing the exercise, and the top-right side (2) shows the data of the specific moment that the therapist has selected. Parts 3 and 4 show the data of the step under supervision. These are presented in graphical manner, which facilitates the supervision of the step by the therapist. Part 5 of the figure contains the input data for the inference engine. These data are collected at the end of each step and are used to obtain the difficulty level of the next step. In addition, part 6 of the figure presents the options for the exercise. The lower part of the interface, part 7, shows the controls that enable the therapist to move forward and backward in steps. This, as shown above, enables the therapist and the patient to perform the exercises independently, without the need of being together in the same place.

## 6. Inference Subsystem

The inference subsystem enables the control of the execution of the rehabilitation exercise without the therapist’s intervention as mentioned above. The presented inference subsystem is divided into two parts. The first part is the inference engine responsible for helping the patient when he/she requires it, namely the FIS for Help (FIS4H). This FIS works while the patient is performing an exercise step and detects the moment when the patient needs to be helped. The second part is the inference engine responsible for adapting the difficulty of the exercise, i.e., the FIS for Difficulty (FIS4D). This FIS is invoked every time the patient completes a step and determines the difficulty of the next step. Next, we will present the two implemented FIS (FIS for Help and FIS for Difficulty) and how they can help the patient to perform the exercises without the constant supervision of a specialist.

### 6.1. FIS for Help (FIS4H)

As mentioned above, this FIS will be used to supervise the execution of the patient’s therapy, detecting when the patient needs help to complete the step and activating the help at that time. This will happen if the patient needs to identify the next action to perform and he/she is unable to do it. Hence, the system itself will be aware of this and act accordingly. The system will always start with the help deactivated, and when the patient needs it, the system will activate the help. Once the patient has selected the target element(s), the system will disable this help again, thus returning to the initial state where no help is provided. This FIS will also help the patient if he/she encounters too many difficulties in the performance of a step. In this case, the FIS will choose to abandon the current step and continue with the next step of the exercise. 

With this control of the exercise execution, we are emulating the supervision of the therapist when a patient is performing an exercise. This system will help the patient, when necessary, and will cancel the exercise if the patient is unable to complete it satisfactorily. This FIS will be invoked every second from the instant the user starts one step of the exercise until the completion of such step. Time between FIS invocations has been set to one second since the Emotiv headset [35], in its community SDK (Software Development Kit) [41], has a refresh rate of one second, and therefore a higher refresh time doesn’t bring any advantage.

#### 6.1.1. Input Data (Antecedents)

Input data is a set of variables that define a specific instant in the exercise’s progress. In this FIS, this data will represent the patient’s current state and how he/she is performing the exercise. Our system uses three multi-valued variables. These variables, as well as their values, have been determined by experts in the field, but they can be modified by therapists to accommodate specific exercise and patient needs. The description of all input variables follows next:

- Current stress (multi-valued): This variable represents, in terms of percentages, the patient’s stress while he/she is carrying out the exercise. This value is used by the system to detect how the patient is feeling. Hence, a high-stress value may indicate that the patient is not comfortable at all with the exercise.

In our case, stress is measured by the Emotiv neuroheadsets [35]. These devices provide several cognitive and emotional metrics, as mentioned above in Section 4.2. One of them is stress, and for this metric it provides a signal that indicate percentage of stress level. In our case, instead of using directly this signal offered by the headset we process it. As a result, a false reading or a peak caused by an occasional incident will not affect the course of the exercise:

(1) $$\;\mathrm{Current}\;\mathrm{stress}=\;\frac{\mathrm{No}.\;\mathrm{high}\;\mathrm{stress}\;\mathrm{samples}}{\mathrm{No}.\;\mathrm{total}\;\mathrm{samples}}\times 100\;$$

The processing we perform consists in taking the last ten readings of the headset (10 s), then classifying these ten values as high or low using a threshold (0.5), and finally applying the formula presented in Equation (1) to these data. The number of readings, as well as the threshold can be modified by the therapist to adjust them to his/her own needs.

**Figure 10:**
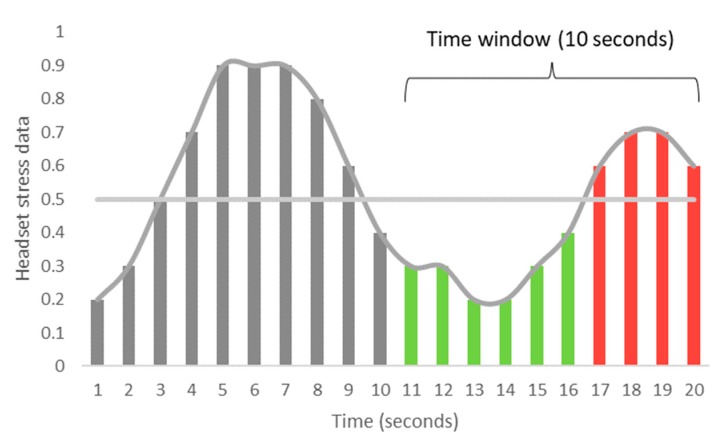
Example of stress processing from the signal obtained from the headset.

An example of the processing of the signal obtained from the headset is shown in Figure 10. On the vertical axis the measurements obtained from the headset are presented, expressed as a decimal. The horizontal axis shows the time of the exercise, in this case 20 s. A horizontal line represents the threshold of 0.5. The last 10 s of exercise execution (seconds 11 to 20) are the time window that our algorithm will take to determine the patient’s stress. The first 6 samples of data obtained from the headset (seconds 11 to 16) do not exceed the established threshold of 0.5, so they are counted as low stress. The next 4 samples (seconds 17 to 20) are equal or above to that threshold, so they are considered as high stress. Applying the Equation (1), the stress level is 40%.

- Time without interaction (multi-valued): This variable considers the time since the user got his/her last successful selection, or since the start of the step (if he/she has not got any successful selection yet). This variable is used to determine the difficulty the patient is experiencing in completing the step at each moment. A high time without interaction could represent a great cognitive difficulty for the patient in finding which the next elements to be selected.
- Consecutive errors (multi-valued): This variable represents the consecutive errors that the patient has had since his/her last item selected correctly or since he/she started the step. Each time the patient succeeds in performing a hit, this variable will return to the value of 0. This variable is also used to determine the problems the patient is having to complete the exercise. A high consecutive errors value indicates that the patient is having trouble to find the next item(s) that should be chosen.

**Figure 11:**
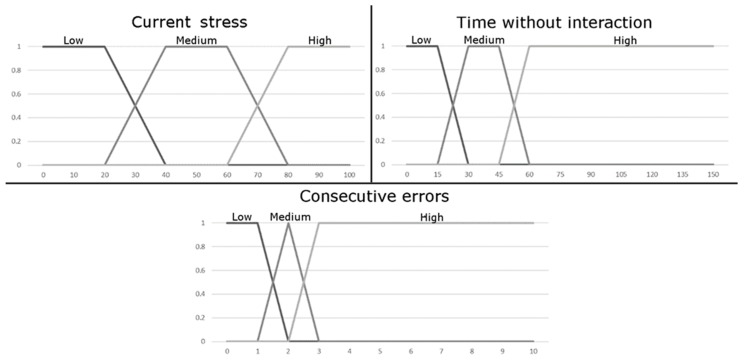
Input values of the FIS4H.

The graphical representation of these inputs’ variables can be found in Figure 11. On the horizontal axis the range of values that input variables can take is displayed. The vertical axis shows the degree of membership, expressed as a decimal. The three values in each graph represent the three considered linguistic tags (Low/Medium/High). In this sense, the charts represent the degree of membership of each linguistic tag to a specific value. The system will consider each of the variables and grant a degree of belonging to each of the linguistic tag. This process, called Fuzzification, will allow the system to know how well each label defines the value, and is used as an input method for the inference engine.

#### 6.1.2. Output Data (Consequents)

In our implementation we have proposed one output variable for the FIS4H. This variable is used to control whether the help system is enabled and if necessary cancel the current step. This output variable is achieved by the system through the Defuzzification method, in which an output variable is obtained from the multiple outputs given by the inference engine from the rules that have been activated. Each of the activated rules will define its output as a trapezoid or triangle that will determine which is the most suitable output for its inputs. Joining all the trapezoids or triangles of the activated rules will create a geometrical shape from which its centroid will be obtained to determine the value of the final output [12].

Because the help will be disabled when the patient selects a correct element, we have not considered the option that FIS may decide to disable help. This avoids loops where the help is continuously activated and deactivated, which can confuse the patient during an exercise. That is, once the help is activated, it will remain active until the patient performs a hit, by selecting the correct element. The output options of this FIS are shown below.

- Do not modify the help: In this case the visual and haptic help will not change from the current value. If the help was disabled, it will remain disabled. This indicates that the patient is doing the exercise correctly. If the help was activated previously, it will remain activated. This indicates that in the near past the system found necessary to activate the help for the patient, but since then the patient has not yet selected the next correct element(s), then the system will continue offering its help.
- Activate help: This system output indicates that the rehabilitation system must activate the visual or haptic help for the patient. This enables the patient to find which is the next element(s) to activate, thus preventing the patient from leaving rehabilitation therapy when facing an exercise that cannot be completed by him her.
- Cancel the step: In the extreme case where the patient is unable to complete the current step, there is an option to cancel the execution of this step. This output will cancel the current step and then the patient continues with the next step, if any, or terminate the exercise if no further steps remain.

#### 6.1.3. Example of a Rule Set

This section shows an example of possible set of rules that the therapist can define for the FIS4H, and the consequences they would have once activated. Table 1 shows the input and output conditions that form the rules of this example set.

- Rule 1 will be activated when the patient has a low stress level. In this case the patient is not stressed by the exercise he/she is doing, so it is not necessary to activate the help and it continues disabled.
- Rule 2 refers to a time when the patient has remained without interaction for a short time and has few cognitive errors. This can happen when the patient starts an exercise, or when he/she has just selected the right element(s), so it is not necessary to activate the help.
- Rule 3 shows a situation where the patient has a high level of stress, but the time without interaction and the consecutive errors have not reached the highest level. In this case, the system will choose to activate the help. This could indicate that the patient is beginning to experience problems in the development of the exercise, and we must act before the situation worsens. This rule is a clear indicator of why it is necessary to use complex rules (with multiple antecedent) to control the execution of the exercise, instead of simple rules (with only one hypothesis). In this case a high degree of stress does not imply that we should cancel the execution of the step or even the exercise, yet it may indicate that the patient is under a high level of stress due to the level of complexity of the exercise. In this case, we suppose that the activation of the help system could offer enough support to select the correct element and reducing his/her stress level. Thus, the use of a single variable, stress in this case, is not enough to determine how the system should behave.
- Rule 4 shows a situation where the patient has not interacted with the system for a long time and has already had some consecutive errors. As in the previous rule, this is an indicator that the patient is facing problems in the development of the step, so it is necessary to help him/her.
- Rule 5 presents an example where the patient may not be able to complete the current step. This may occur because the current step is too complex for the patient, or it may even mean that the current step is wrong, meaning that the therapist has made a mistake in its design. A high level of stress, a high time without interaction and high consecutive errors are the conditions that must be met for activating this output option, and as a result the system will choose to cancel the step and proceed to the next one.

### 6.2. FIS for Difficulty (FIS4D)

As described above, this FIS is responsible for adapting the difficulty of the exercise, selecting the level of difficulty of the next step to be performed by the patient. The therapist has defined different difficulty levels for each of the steps described, as shown in Section 3.3. In this sense, a multi-step rehabilitation exercise can be performed multiple times, thus offering a unique and personalized level of difficulty to each patient at any given time. Hence, we are fostering the replayability [42] of the developed exercises. This FIS will attempt to implement the knowledge that a therapist has when supervising the development of an exercise, modifying the difficulty of the exercises depending on the performance of the patient while completing the previous steps, as well as the previous rehabilitation sessions. The FIS will be called each time the patient completes a step or completes the exercise, and it will take information on how the previous step was performed and how the patient was doing while performing it. If the patient did not have any problems while performing the exercise, it will mean that the difficulty level of exercise is not enough for the patient, and it will be decided to increase this difficulty. However, if the patient has had problems with exercise, it may mean that the therapist has overestimated the patient’s cognitive abilities, and therefore, the next step of exercise will be easier than the one just performed. It is also considered a mid-point where the level of difficulty is appropriate for the patient’s cognitive conditions, and therefore the choice will be to leave the difficulty unchanged.

#### 6.2.1. Input Data (Antecedents)

In this FIS4D, the input data represents the patient’s performance in the previous step, as well as the patient’s state in the last moments of the exercise. In our system, we use two multi-value variables and one binary variable, which makes a total of three variables. As in the previous FIS, these variables, as well as their values, have been determined by specialists in the field. Moreover, as highlighted above, these variables can be adapted by rehabilitation specialists to suit their specific needs, and those of their patients. The description of all input variables follows next:

- Step average stress (multi-valued): This variable, as in the FIS4H, represents under what level of stress the patient is. Although in this case the level of stress does not reflect a specific point, it reflects the average level of stress during the last part of the exercise. By default, the values of the last quarter of the exercise (25%) will be taken and the average will be calculated, but this percentage of the exercise can also be modified by the therapists. This value has been set to a quarter as it is large enough to avoid peaks of stress that may occur in the last moments of the step, but it is a small enough to avoid the stress changes that may occur at the beginning of the step, when the patient begins to explore the environment.
- Step errors ratio (multi-valued): This variable represents how many errors, with respect to the total of actions (errors + hits) the patient has made in the previous step, as shown in Equation (2). This tells us how accurate the patient has been in the previous step regardless of the number of total errors. This enables us to create rules that include short exercises, where the number of elements to be selected is small (therefore the errors will usually be few), and long exercises where the number of elements to be selected is large (therefore the errors will usually be more than in the previous case):
  (2) $$\;\mathrm{Step}\;\mathrm{errors}\;\mathrm{ratio}=\frac{\mathrm{No}.\;\mathrm{errors}}{\mathrm{No}.\;\mathrm{total}\;\mathrm{actions}}\;$$
- Step canceled (binary): This variable will indicate to the system whether the previous step was completed successfully or whether the FIS4H had to cancel the execution of the previous step. In the event that the previous step has had to be cancelled, it is necessary to decrease the difficulty of the exercise, as this will indicate that the patient has had serious problems in that previous step. This variable is binary and can only take the values of true, if the previous step has had to be cancelled, or false, if the user has been able to complete the previous step correctly.

**Figure 12:**
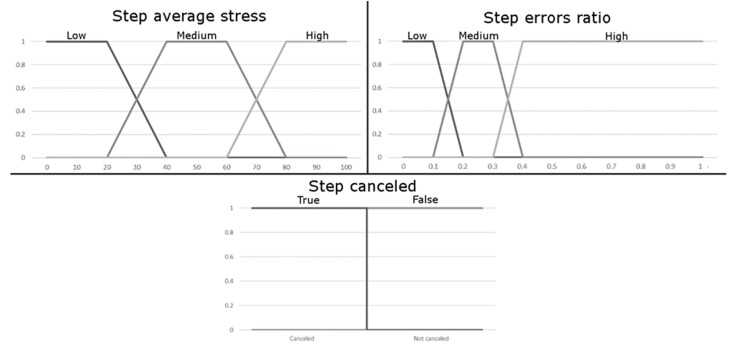
Input values of the FIS4D.

The information for the variables is shown in Figure 12. On the horizontal axis the range of values that input variables can take is displayed, and the vertical axis shows the degree of membership, expressed as a decimal. The three values in each graph represent each of the linguistic tags considered (Low/Medium/High). In this sense, the graphs represent the degree of membership of each linguistic tag to a specific value. As in the previous case, the values represented in this graphic allow the system, through the fuzzification process, to determine the input values of the inference system.

#### 6.2.2. Output Data (Consequents)

In our system we consider a single output variable, which can take five linguistic labels. These output variables are obtained using the same Defuzzification method as in the previous FIS. These values will tell the system what the difficulty of the next step will be. The output options of this FIS are shown below.

- Difficulty + +: This output indicates to the system that the difficulty must be increased significantly, so that the next step will have a difficulty two levels higher than the baseline.
- Difficult +: In this case the next difficulty should be a little higher than the base difficulty, so the difficulty will be raised one level above the base.
- Difficulty =: When the system obtains this output, it indicates that the difficulty of the exercise is appropriate for the patient, so it will not modify it, and the difficulty obtained will be equal to the base difficulty.
- Difficulty −: This output indicates that the difficulty should reduce a little, so the system will have to decrease the difficulty of the next step one level below the base difficulty.
- Difficulty − −: In this case the patient has had serious problems completing the previous step, and therefore the difficulty of the next step should be reduced to two levels.

If the FIS wants to reduce the difficulty of the next step, but cannot reduce it more, the system will choose the lowest difficulty. Similarly, if the FIS attempts to increase the difficulty of the next step, but there is no greater difficulty, the system will choose the higher difficulty.

#### 6.2.3. Example of a Rule Set

This section shows an example of possible set of rules for the FIS4D, and the consequences they would have once activated. Table 2 shows the input and output conditions that form the rules of this example set.

- Rule 1 shows a situation where the patient has completed the previous step with a low error rate. This indicates that it is possible to greatly increase the difficulty of the exercise. So, the output of the FIS will be *Difficulty + +.*
- Rule 2 is activated when the patient’s average stress during the last part of the exercise is low. This indicates that the patient has not experienced difficulties in the completion of the last step, so the next step may be more difficult. In this case the output of the FIS will be *Difficulty +.*
- Rule 3, the patient has experienced moderate stress and has made a medium number of errors. Therefore, it is recommended not to increase the difficulty, but there is no reason to decrease it either, so the difficulty will remain the same (*Difficulty =*).
- Rule 4 is obtained when the user has made many errors. The high difficulty of the previous step has caused the patient to make many errors, so the most convenient thing to do is to reduce the difficulty. According to this the output of the FIS will be *Difficulty* −.
- Rule 5 will be met when the patient has not successfully completed the previous step, that is, when the FIS4H has determined that it is recommended that the step be cancelled. In this case it is better to considerably reduce the difficulty of the exercise (*Difficulty* − −).

#### 6.2.4. Example of Execution

**Figure 13:**
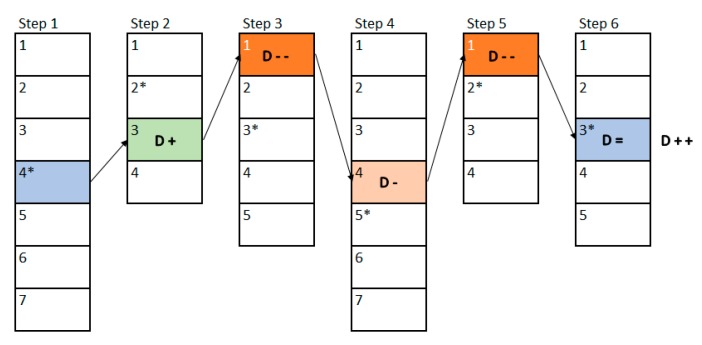
Example of the first execution of an exercise and modification of the difficulty performed by the FIS.

In this example, we will use the same exercise presented in Section 3.3, which was composed by six steps. Figure 13 shows the first execution of the exercise and the changes in difficulty that have been made between the steps of it. The vertically-stacked rectangles represent each step of the exercise (six in this example), and each division inside the rectangle represents a level of difficulty created by the therapist (the first step has seven levels, the second four, and so on). The numbers within each step represent the relative difficulty in that step, being number 1 the easiest one, and getting more difficult as the number increases. The difficulty level that the therapist assigned as the base level in the design phase has been represented with an asterisk.

The patient will begin to perform the exercise in the first step and in the 4th difficulty level of this step, since this has been established by the therapist through the base level. Once the first step of the exercise has been completed, the FIS indicates that the difficulty must be increased a little (D +), so in the second step a difficulty of 3 must be established, which is the base level (2) plus the increase established by the FIS (1). Once the second step is completed, the FIS indicates that the difficulty must be greatly reduced (D − −), so that a difficulty level of 1 is obtained. In the third step the output of the FIS is that the difficulty must be lowered a little (D −), so that level 4 is reached. Once the fourth step is completed, the output of the FIS is to lower the difficulty considerably (D − −), as the next step cannot decrease two levels from its base level, the difficulty that will be shown will be the lowest (1). In the fifth step the result is to maintain the same difficulty, so the difficulty of the last step will be the base one, as proposed by the therapist. Finally, once the patient has completed the last step, the FIS is called again, and the result is that the difficulty should be increased considerably (D + +), so that the next time the user performs the exercise he will no longer start with difficulty 4 of the first step but will do it from two more levels (from level 6).

**Figure 14:**
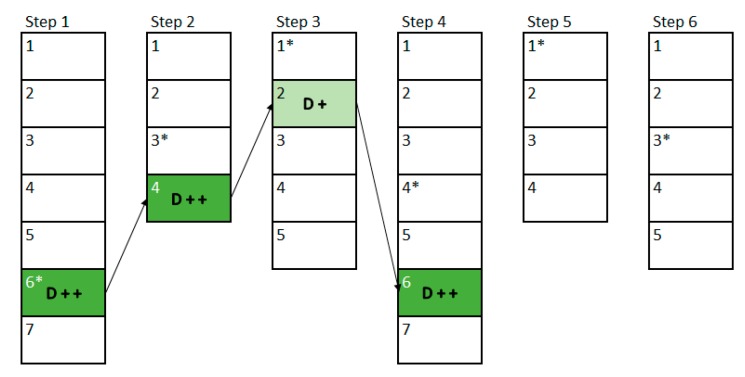
Example of the second execution of a program and modification of the difficulty performed by the FIS.

Figure 14 shows an example of a second execution of a patient’s rehabilitation exercise. As it can be seen in comparison to Figure 13, the initial difficulties have changed from those the therapist established at the design phase. In the first step this new base level is established according to the final output of the previous interaction, in this case the previous interaction showed that the level had to be increased by 2, so the base level of difficulty has changed from 4 to 6. In the rest of the cases, the base level is established according to the level of difficulty that was performed in the previous interaction.

In this second interaction, the variation in difficulty follows the same rules as those shown in the previous case. But, in this one, the rehabilitation exercise has not been completed, so the next time the patient resumes his/her rehabilitation exercise he/she will start from the point where he/she left. In this case, the patient has completed the first three steps of the exercise, and the fourth step has either not been started or has been interrupted, so the next time the patient resumes his/her rehabilitation, he/she will be start in step 4 directly. In this sense, by controlling the difficulty in each step of the exercise, the patient can ‘re-play’ his/her rehabilitation exercises. Therefore, the patients will get a unique experience in their rehabilitation, which also fits their personal rehabilitation needs. This also encourages them to self-improve, which leads to a more bearable rehabilitation for patients and their relatives.

### 6.3. Construction of Rules

**Figure 15:**
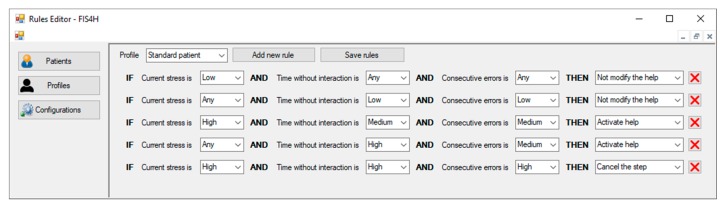
Rules editor interface of the FIS4H.

As the system will be used by non-computer expert, it is relevant to offer an adequate interface for supporting the fuzzy rules entry task. Since there are two inference engines in our proposal, we have also defined two interfaces to manage the rules of each FIS. In Figure 15 we present the rules editor interface of the FIS4H, in which the rules built in Section 6.1.3 are displayed. As it can be seen, the therapists can create several profiles related to group of patients with similar features. Moreover, they can adapt this profile to a specific user by choosing the value of each fuzzy variable.

## 7. Evaluation

A fundamental factor of rehabilitation technology research is knowing how therapists or end users react to new technology proposals [43,44]. It is important that the design and development of telerehabilitation systems consider user-centered approaches [45], especially to find out if the proposed system fits the needs and preferences of the end user, as well as the context in which it can be implemented [9,46]. Some studies have shown that the rate of abandonment of technological systems in the health field is 40% [47]. In this sense, an experimental task has been developed, in which experts in rehabilitation, occupational therapists, physiotherapists, physicians and psychologists provide their opinion in order to know the satisfaction with the proposed telerehabilitation system.

### 7.1. Experimental Context

The main goal of this experiment, defined by using Goal Question Metric [48] is: analyze the proposed assistive cognitive rehabilitation system for the purpose of potential users’ satisfaction, for physical and cognitive rehabilitation therapists in the context of occupational therapists, physiotherapists, physiologists, medicine doctors and physiotherapy students. With this aim, Table 3 presents the hypothesis that this experiment tried to demonstrate.

### 7.2. Design

We developed a demonstration video to show the use of the telerehabilitation tool for therapists. The length of the video was 13 min, and it took approximately 5 min to complete the questionnaire (see Table 4). The total duration of each assessment session with each expert ranged from 30–35 min. The video showed how a rehabilitation expert used the tool in each phase, as well as how the therapist used it to (1) login; (2) create an exercise; (3) assign an exercise to the patient; (4) use the sensors and actuators; (5) performed the exercise; and (6) checked the execution and performance of session. After watching the video, each expert completed the questionnaire. The survey consisted of 16 five–point ratings scales (1: Strongly disagree to 5: Strongly agree). In addition, after each session the evaluator noted all the observations and comments made by each expert. All participants gave their informed consent to be included in the study. No personal data that could identify them was collected, guaranteeing the anonymization of the databases. All questionnaires were conducted by a neuropsychologist and an occupational therapist in a quiet room at the Health Science Faculty in the University of Granada. All data was collected during the month of July 2018.

### 7.3. Data Analysis

Descriptive statistics were used for qualitative and quantitative variables, the socio-demographic questionnaire and satisfaction with the telerehabilitation system. The frequencies and percentages have been calculated for each of the categories of the qualitative socio-demographic variables. In order to know the satisfaction of the therapists after their exposure to the telerehabilitation system, the mean scores and the standard deviations obtained in each item of the questionnaire and in the overall satisfaction were analyzed. The corresponding confidence intervals were also calculated (95% CI). The t-test was also used to analyze the differences in the three dimensions of satisfaction studied: satisfaction with the rules (RUL), satisfaction with the sensors (SEN) and satisfaction with the monitoring display (MON). The level of statistical significance was set to a value of *p* < 0.01. All data were analyzed with IBM 24 SPSS software.

### 7.4. Results

In this section we will show the statistical data of the participants, as well as the results obtained from the questionnaires, all this to conclude with the answers to the proposed research questions.

#### 7.4.1. Characteristics of the Therapists Sample

Participation was voluntary, and selection was made on the basis of a convenience, non-probabilistic sample. Data on the characteristics of 20 therapists were extracted from the questionnaires and displayed in Table 5. 65% of the experts were women and 75% were occupational therapists, who are usually responsible for cognitive rehabilitation. Most of them work in public rehabilitation centers (55%). The most frequent pathology they worked with was people with acquired brain damage (40%), followed by older people with disabilities (25%). 

#### 7.4.2. Expert Satisfaction Questionnaire Score

To obtain a first result from the expert satisfaction, the average results for each one of the questions was computed. As it can be seen in Table 6, except for question Q14 (“Thanks to the results viewer I do not consider my presence to be necessary during the rehabilitation session”), for all of them it was obtained a result higher than 4 in a scale from 1 to 5. Therefore, we can intuit that the satisfaction of the experimental subjects with the proposal was high. Moreover, these positive results are also supported by the final question (“Overall satisfaction with the proposal”), where an average result of 8.76 in a scale from 0 to 10 (σ = 1.70) was obtained.

**Figure 16:**
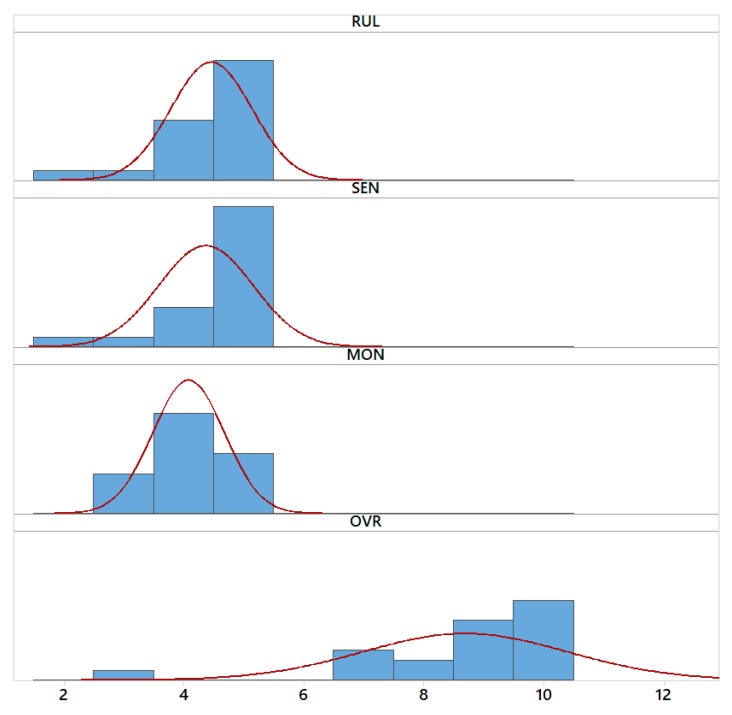
Distribution of data for the four dependent variables.

Additionally, in order to accept or reject our null hypothesis (Table 3), we performed a deeper statistical analysis. Hence, the answers of each participants for the three first dependent variables (RUL, SEN, MON) were grouped by computing their average value. Then, with these three variables, along with the last one (OVR), which was obtained directly from the final question, we performed a series of t-tests [49,50]. The results of such tests can be seen in Table 7 and Figure 16.

Considering the results shown in Table 7, we can conclude that the average is higher than 3 for the dependent variables RUL, SEN and MON, and greater than 5 for OVR, with the 0.05 level of confidence. Because of that reason, we accept our four null hypotheses. Therefore, potential users’ satisfaction with rule-based system, used sensors and remote monitoring of the session is higher than 3 in a scale from 1 to 5, and their overall satisfaction with the proposal is higher than 5 in a scale from 0 to 10.

### 7.5. Results Breakdown

One of the elements most valued by the therapists was the remote exercise viewer and the information that allowed to know about the execution of the patient in each exercise, as well as the possibility of observing the patient’s evolution. Occupational therapists indicated the interest in collecting the recording of the patient’s performance, especially in those with anosognosia and unilateral neglect where video feedback is a very useful strategy in cognitive rehabilitation [51,52]. In addition, therapists can check on their application what kind of mistakes the patient commits, such as losing the sequence, changing the order of the task. Moreover, it can be observed whether the patient is aware of the mistakes he/she makes, and whether he/she tries to correct them. In this sense, the therapist application can be a tool that enables them to know if the patient shows an adequate attentional control while performing the activity. Because of these characteristics, the therapist can develop a clinical reasoning about the course of treatment, the strategies that the patient uses, and which of them are more beneficial to establish new goals. In this sense, it is a very useful tool to analyze the errors of those patients who may have an executive dysfunction and patients with cognitive impairment or dementia, where this type of deficit affects their personal autonomy and daily performance [53].

The second element most valued by the experts was the possibility of offering different types of help according to the needs of the patients. In particular, the most appropriate help can be used according to the learning strategies followed and the type of signals that are easiest to process for each patient (visual, auditory or haptic), thus optimizing the rehabilitation results. This type of information is essential for planning and executing treatments for patients with acquired brain damage. This is because it is possible that the processing of some type of sensory modality may be compromised. Therefore, different types of sensory stimuli may be necessary to facilitate the re-learning of new skills or to rehabilitate cognitive or motor functions [54].

Thirdly, it is important to highlight the positive evaluation made by therapists, who stated that this is a tool that enables the patient’s level of difficulty to be continuously adapted and modified according to their performance. Fourth, and in relation to the previous point, the stress sensor was also highly valued, especially at the beginning of the exercise, since it is known that if the activity that the patient faces is very complicated or different from the ones he/she already knows, it generates a stress situation for the patient. If so, it is very likely that the patient will not be motivated to carry out the therapeutic activity, so he/she will abandon it, thus not having a good adherence to the treatment [55].

Another aspect that occupational therapists highlighted was the potential of the tool to record and systematize treatments. This aspect was mostly well valued by recent graduated therapists, as it could help them in their clinical reasoning, based on the results viewer and the system of rules [56]. The possibility of grading the complexity of the exercise or therapy and the autonomy that this system provides them with was also highly valued. Hence, the transfer of learning can be graduated from a simpler task to more complex ones with greater cognitive demands and greater ecological value, as happens in the activities of daily life [56,57,58,59]. On the other hand, it is worth noting that Q14 (“Thanks to the results viewer I do not consider my presence to be necessary during the rehabilitation session”) was the least rated question. The possible explanations for this fact will be dealt with in the following section.

### 7.6. Limitations

The study, which was conducted with experts, has some limitations. First, the experts were selected by non-probabilistic convenience sampling. This may limit the extrapolation of the results, although their usefulness in exploratory studies such as this one has been demonstrated [60]. Other limitation is the limited sample size of participants. In this study, the sample of 20 participants may be small for most studies. We have managed this threat by using expert therapists with knowledge of computer-assisted rehabilitation systems as well as in rehabilitation techniques that are implemented by the evaluated system. This has enabled us to reduce the sample of participants without having a negative influence on the data obtained in the experiment. Also, we would like to point out that the profile of occupational therapists with expertise in cognitive rehabilitation with people with brain damage and cognitive impairment in the elderly makes their opinions and participation very useful for the study of the proposed tool.

Another limitation of the study is the fear of replacement of rehabilitation experts by machine automation. The system under assessment attempts to offer greater freedom to both patients and therapists, but the later may see this development as a threat to their jobs and therefore evaluate it negatively in the study. We have addressed this threat through two ways. The first one was to enroll therapists who are experts in computer-assisted rehabilitation systems, who already know the benefits of such systems. They knew these systems are not designed to replace them, but to make the work easier for them and for patients. The second way to deal with this problem, has been to explain to the participants before the experiment the real motivation of the system, thus clearing up any doubts about this aspect. However, in question Q14, which refers to this limitation, it has been possible to see how a relevant number of participants show some disagreement regarding the usefulness of the system for their use without the need for the therapist to supervise the rehabilitation session. 

One shortcoming of this study is that we did not gather additional information that could explain why the therapist gave a low score in Q14. In future studies, this question should be addressed to know if this low score could be related, in part, related to a perception by therapists that they are irreplaceable. More studies are needed to know if these may be due to some type of fear regarding the therapist’s application, thus making it necessary to improve the system. In future studies it would be useful to add some additional question, in the event of having rated with a score less than or equal to 3 in Q14. This result could be due to different reasons that should be investigated. One possible explanation could be that the therapists feel that their work cannot be completely replaced and that the system does not offer enough autonomy and needs to be improved. It is also possible that this could be due to the fear of being dispensable [47,61]. According to Perlusz [62], this type of emotional processes is not found in the most commonly used technology-acceptance models and it would be interesting to take it into account in future studies on behavioral intention.

## 8. Conclusions and Future Work

In this work, we have presented a new version of our previous cognitive rehabilitation system [17,18] for enabling at-home rehabilitation (telerehabilitation). This new proposal has focused on the autonomy of the therapist and the patient, enabling them to carry out the rehabilitation tasks whenever and wherever they want. With this aim, an inference system has been added, that provides higher intelligence to the system. Besides, this system enables people without specific knowledge in cognitive rehabilitation, such as patient’s relatives or caregivers, to help patients perform exercises. This increases the number of patients who can be treated by rehabilitation specialists and enables cognitive rehabilitation for people who previously did not have access to this type of rehabilitation.

At the beginning of this paper, the architecture of the developed system was presented, showing the therapist’s and patient’s application, as well as the help and difficulty control systems. Later, the sensors and actuators used were presented, in addition to the functionality of each one, with special reference to the limitations they have, and the solutions proposed to avoid these limitations. Afterwards, the remote exercise viewer was shown, which enables the therapist to supervise the exercises performed by the patient. Then, the paper has focused on the design of the inference system. Our system implements two different FIS, being the first one invoked during the exercise and controls the activation of the help for the patient, and the other one that is called each time the patient finishes a step of the exercise and controls the difficulty of the next step.

After the presentation of the system, the evaluation by the experts was shown. The experts provided such a favorable evaluation in all areas of the system (rule-based, sensors, remote monitoring and overall). The most important elements are the remote results viewer system, the possibility of offering different types of help to the patient, as well as the modification of the difficulty to adapt the exercise to the patient’s needs by using the stress data collected by the EEG headset. However, in the evaluation of the proposal, question Q14 (“Thanks to the results viewer I do not consider my presence to be necessary during the rehabilitation session”) received the lowest score. This, as discussed in Section 7.5, may be because therapists think they would need system improvements to enable proper telerehabilitation, or it may be due to fear of being dispensable. More studies are needed in this area to determine which is the correct statement.

In conclusion, our cognitive rehabilitation system provides greater autonomy for both therapists and patients. On the one hand, the therapist can leave much of his/her expertise to FIS, so that it can control the execution of an exercise by increasing/decreasing its difficulty, activating/deactivating the help, or even stopping the steps if they are too complicated for the patient. On the other hand, it also provides autonomy for the patient, who can perform the exercises without the need for constant supervision by a cognitive rehabilitation specialist. This enables the patient to perform this therapy in rehabilitation centers where there are several simultaneous patients (more than currently possible) or even at home.

As future work, we will work on modifying the haptic device to make it easier to wear. In addition, further evaluations with rehabilitation experts will be proposed to determine the appropriate answer to question Q14, proposing improvements to the system, the sensors, the FIS or any other aspect that the experts consider appropriate. Also, we would explore the user of other physiological parameters of the patience as an input to the inference system, which allow to determine with greater precision which is the true state of the patient. One of the variables to consider may be physical or mental fatigue, which may be more relevant over long periods of rehabilitation. Another proposed future work is to add new functions that allow the patient to ask for help when he/she needs it. This added functionality would allow user, or their families or caregivers, to have more control over the development of rehabilitation therapy, allowing them to be helped when patients think that they need it. On the other hand, it would allow the system’s database to be completed automatically based on patients’ real needs, allowing the therapist to spend less time creating, modifying and fine-tuning rules for patients. Besides, the last proposed future work is to conduct an evaluation with potential patients, which allows us to determine the final adequacy of the system to its final purpose. It would be interesting to carry out future studies about the effectiveness of treatment with a randomized controlled trial (RCT), in which a group of patients receive treatment first without the system and only conventional therapy, and another group to start the treatment with the telerehabilitation system and then compare the results and objectives of them.

Finally, different exercises will be developed based on the activities of daily life, both basic and instrumental, that will enable the patient not only to develop a certain cognitive and/or physical component, but also to integrate his/her skills and abilities into a functional activity. This will enable him/her to advance towards the highest degree of personal independence, autonomy and participation in the activities of daily life [57,58,63]. For this purpose, activities which are contextualized in the real life of the patient will be used (e.g., at home, etc.).

## Figures and Tables

**Table 1:** Example rule set of the FIS4H.

Nº	Current Stress	Time without Interaction	Consecutive Errors	Output
1	Low	Any	Any	Do not modify the help
2	Any	Low	Low	Do not modify the help
3	High	Medium	Medium	Activate help
4	Any	High	Medium	Activate help
5	High	High	High	Cancel the step

**Table 2:** Example rule set of the FIS4D.

Nº	Step Avg. Stress	Step Errors Ratio	Step Canceled	Output
1	Any	Low	Any	Difficulty + +
2	Low	Any	Any	Difficulty +
3	Medium	Medium	Any	Difficulty =
4	Any	Hight	Any	Difficulty −
5	Any	Any	Yes	Difficulty − −

**Table 3:** Main features of the experiment.

**Null Hypothesis**	H_0A_: Potential users’ satisfaction with rule-based system is higher than 3 in a scale from 1 to 5. H_1A_: ¬H_0A_
	H_0B_: Potential users’ satisfaction with used sensors is higher than 3 in a scale from 1 to 5. H_1B_: ¬H_0B_
	H_0C_: Potential users’ satisfaction with remote monitoring of the session is higher than 3 in a scale from 1 to 5. H_1C_: ¬H_0C_
	H_0D_: Potential users’ overall satisfaction with the proposal is higher than 5 in a scale from 0 to 10. H_1D_: ¬H_0D_
**Dependent Variables**	Potential users’ satisfaction with rule-based system (RUL)
	Potential users’ satisfaction with used sensors (SEN)
	Potential users’ satisfaction with remote monitoring of the session (MON)
	Potential users’ overall satisfaction with the proposal (OVR)

**Table 4:** Expert Satisfaction Evaluation Items.

Category	Question
Rule-based system	Q1. The variables that appear in the rules are adequate
	Q2. The variables’ labels are adequate
	Q3. The rule-based system can capture my knowledge about how to adapt each exercise
	Q4. The help-activation system is suitable for this type of rehabilitation
	Q5. The difficulty-adaptation system improves the rehabilitation
Used sensors	Q6. The used sensors collect the patient’s information required to perform these exercises
	Q7. The information collected by the EEG sensor for stress detection is of interest for the treatment of the patient
	Q8. The haptic stimulator helps to carry out the exercises
	Q9. The sensors do not limit the movement of the patient
	Q10. The system can be use easily by patients with the assistance of a caregiver/family
Remote monitoring	Q11. The activity viewer helps me follow the evolution of the exercise
	Q12. The patient’s results viewer is straightforward
	Q13. I can get all the data I need through the results viewer
	Q14. Thanks to the results viewer I do not consider my presence to be necessary during the rehabilitation session
	Q15. The information on the result viewer is displayed appropriately
Other	Overall satisfaction with the proposal

**Table 5:** Experts’ profiles.

Characteristic		N	Percentage (%)
Gender	Male	7	35
	Female	13	65
Specialty	Occupational Therapist	15	75
	Physical Therapist	3	15
	Others	2	10
Type of practice	Public Hospital	11	55
	Private Clinic	5	25
	Combined	4	20
Field of Practice	Brain Injury	8	40
	Geriatric & Gerontology	5	25
	Neurodegenerative diseases	2	10
	Other fields	5	25
Employment status	Part-Time	3	15
	Full-Time	17	85
Experience with Telerehabilitation Systems	Yes	6	30
	No	14	70

**Table 6:** Mean and standard deviations result for experimental questions.

Category	Rule-Based System					Used Sensors					Remote Monitoring				
Question	Q1	Q2	Q3	Q4	Q5	Q6	Q7	Q8	Q9	Q10	Q11	Q12	Q13	Q14	Q15
$\bar{x}$	4.40	4.35	4.35	4.65	4.60	4.40	4.25	4.60	4.40	4.25	4.75	4.00	4.25	3.05	4.40
***σ***	0.88	0.87	0.93	0.74	0.68	0.82	1.16	0.82	1.09	0.96	0.55	0.80	0.72	0.94	0.88

**Table 7:** Results of statistical tests.

Dependent Variable	Sample Size	Mean	90% Confidence Interval	Standard Deviation	Target	*p*-Value	α
RUL	20	4.47	(4.15–4.78)	0.67	3 (1 to 5)	<0.001	0.05
SEN	20	4.38	(4.01–4.75)	0.79	3 (1 to 5)	<0.001	0.05
MON	20	4.94	(4.60–5.28)	0.73	3 (1 to 5)	<0.001	0.05
OVR	20	8.70	(7.90–9.50)	1.72	5 (0 to 10)	<0.001	0.05
